# Integrated multi-modality image-guided navigation for neurosurgery: open-source software platform using state-of-the-art clinical hardware

**DOI:** 10.1007/s11548-021-02374-5

**Published:** 2021-05-03

**Authors:** Jonathan Shapey, Thomas Dowrick, Rémi Delaunay, Eleanor C. Mackle, Stephen Thompson, Mirek Janatka, Roland Guichard, Anastasis Georgoulas, David Pérez-Suárez, Robert Bradford, Shakeel R. Saeed, Sébastien Ourselin, Matthew J. Clarkson, Tom Vercauteren

**Affiliations:** 1 School of Biomedical Engineering and Imaging Sciences, King’s College London, London, UK; 2 Wellcome/EPSRC Centre for Interventional and Surgical Sciences, UCL, London, UK; 3 Department of Neurosurgery, National Hospital for Neurology and Neurosurgery, London, UK; 4 Centre for Medical Image Computing, UCL, London, UK; 5 Department of Medical Physics and Biomedical Engineering, UCL, London, UK; 6 Research Software Development Group, Research IT Services, UCL, London, UK; 7 The Ear Institute, UCL, London, UK; 8 The Royal National Throat, Nose and Ear Hospital, London, UK

**Keywords:** Image-guided surgery, Neurosurgery, Intraoperative ultrasound, Intraoperative neuromonitoring, Open source, Computer-assisted interventions

## Abstract

**Purpose:**

Image-guided surgery (IGS) is an integral part of modern neuro-oncology surgery. Navigated ultrasound provides the surgeon with reconstructed views of ultrasound data, but no commercial system presently permits its integration with other essential non-imaging-based intraoperative monitoring modalities such as intraoperative neuromonitoring. Such a system would be particularly useful in skull base neurosurgery.

**Methods:**

We established functional and technical requirements of an integrated multi-modality IGS system tailored for skull base surgery with the ability to incorporate: (1) preoperative MRI data and associated 3D volume reconstructions, (2) real-time intraoperative neurophysiological data and (3) live reconstructed 3D ultrasound. We created an open-source software platform to integrate with readily available commercial hardware. We tested the accuracy of the system’s ultrasound navigation and reconstruction using a polyvinyl alcohol phantom model and simulated the use of the complete navigation system in a clinical operating room using a patient-specific phantom model.

**Results:**

Experimental validation of the system’s navigated ultrasound component demonstrated accuracy of $<4.5\,\hbox {mm}$ and a frame rate of 25 frames per second. Clinical simulation confirmed that system assembly was straightforward, could be achieved in a clinically acceptable time of $<15\,\hbox {min}$ and performed with a clinically acceptable level of accuracy.

**Conclusion:**

We present an integrated open-source research platform for multi-modality IGS. The present prototype system was tailored for neurosurgery and met all minimum design requirements focused on skull base surgery. Future work aims to optimise the system further by addressing the remaining target requirements.

## Introduction

Image-guided surgery (IGS) has become an indispensable tool in the management of brain tumours. IGS and the use of neuronavigation allow for smaller, more precisely positioned incisions and the accurate localisation of tumours and surrounding structural and functional regions which may be at risk during surgery [10]. However, current clinical neuronavigation systems are limited by their inability to account for intraoperative brain shift encountered during surgery.

Intraoperative ultrasound (iUS) is a portable system offering real-time imaging and has become an increasingly popular tool within neurosurgery due to its comparatively low cost and real-time feedback [5, 18]. Unlike the acquisition of iMRI, the use of iUS is easily incorporated into the surgical workflow [3, 22]. Several different ultrasound systems have been reported in neurosurgery including the SonoWand (SonoWand, Mison Trondheim, Norway) [5, 29], Sonosite M Turbo (SonoSite Inc, Bothell, WA) [17], Aloka SSD 3500 (Aloka-Hitachi, Wiesbaden, Germany) [1] , Sonoline Omnia (Siemens, Erlangen, Germany) [12], Capasee II (Toshiba, Tochigi Ken, Japan) [17] and the BK Ultrasound system (BK Medical, Peabody, MA) [13] and most have focused on the use of iUS in neuro-oncology [15, 21].

However, despite this previous work, iUS remains an under-utilised tool in neurosurgery: firstly, because neurosurgeons are not very familiar with US as an imaging modality and, secondly, because US is typically acquired and visualised in unfamiliar planes. This limitation may be overcome by the integration of iUS images with neuronavigation. Some commercially available neuronavigation systems do have the capability to integrate image-registered intraoperative three-dimensional ultrasound (i3DUS) with neuronavigation (e.g. Brainlab system^1^ and Esaote system^2^), but no system permits the integration of additional intraoperative monitoring modalities such as continuous intraoperative neurophysiolgical monitoring and stimulation. Such a capability would be particularly useful in neuro-oncology and skull base neurosurgery.

In skull base neurosurgery, it is vital to know exactly where critical neural structures such as the facial nerve are located in order to minimise nerve injury and post-operative morbidity. Intraoperative neurophysiological stimulation is the current standard to detect the facial nerve intraoperatively, but current methods are not integrated with neuronavigation and do not provide a means to visualise points of stimulation on the patient’s imaging. Skull base neurosurgery, and neuro-oncology neurosurgery in general, would benefit from a fully integrated navigation system combining preoperatively acquired MRI and CT images, volumetric representation of the tumour and surrounding functional anatomy (e.g. cranial nerves), i3DUS and navigated intraoperative neurophysiological monitoring and stimulation.

Most commercially available neuronavigation systems such as the Medtronic Stealthstation and Brainlab systems are closed systems, and their image data, algorithms and visualisation methods are typically not easily accessible to research groups. As such, various open access IGS and image-guided therapy (IGT) systems have been developed by the medical and research communities including 3D Slicer [8], NifTK [7], MITK [19], IBIS [16] and CustusX [2]. 3D Slicer and MITK are generic medical imaging research platforms that when combined with plugins such as the SlicerIGT module and the PLUS Toolkit can provide integrated navigation and ultrasound imaging. IBIS and CustusX are both open-source research platforms designed for neurosurgery, but integrating these with commercially available navigation systems is non-trivial, and there is limited scope for integrating iUS with additional functionalities such as neuromonitoring.

We describe the development of an open-source multi-modality IGS platform (available at https://github.com/UCL/SkullBaseNavigation), designed to integrate with a commercially available neuronavigation system. As a proof of concept, the system was designed for skull base surgery although it could be used during any cranial neuro-oncology surgery where multi-modal intraoperative guidance is desired.

## Design requirements

In this section, we present design requirements for an integrated intraoperative imaging and navigation system for skull base surgery, named the Skull Base Navigation [SBN] system. Following the assumption that the system should be compatible with the neuronavigation and ultrasound systems typically used at our institution (Medtronic Stealthstation and BK 5000 Ultrasound systems, respectively), Table 1 provides an overview of the design requirements (**R** X ). These include: (1) requirements imposed by the clinical environment in the operating room (OR) during surgery; (2) requirements desired by the operating surgeon; and (3) specific technical requirements needed for the purpose of intuitive real-time surgical navigation.

Requirements imposed by the clinical environment were established through consultation with surgical team members and an understanding of medical device regulations.^3^ The system’s key functional requirements were identified by an experienced team of Neurosurgeons and Otolaryngologists (JS, SRS and RB) familiar with using the stand-alone commercial neuronavigation, neuromonitoring and ultrasound systems. Specific technical requirements were then established in order to meet these functional requirements.

To aid development, a minimum and target requirement is provided.

**Table 1:** Design requirements for an integrated skull base navigation system

Requirement	Minimum requirement	Target requirement
R1. System assembly	Surgical hardware should comprise of standard clinical devices. Assembly should be straightforward and achievable without technical support. It should not impede routine surgical workflow	Ibid Ideally should be completed within 15 min
R2. Surgical safety	Intraoperative system components must not be altered from their designated use and methods of maintaining intraoperative sterility must comply with standard clinic practice	Ibid
R3. Ultrasound probe calibration	Intraoperative system calibration should not impede surgical workflow (completed in less than 1 min and should achieve satisfactory spatial accuracy	Pre-calibrated ultrasound transducers eliminating user calibration achieving detailed spatial accuracy
R4. Image calibration	Fixed image calibration at 4.5 cm image depth	Variable image calibration that automatically updates depending on the image depth
R5. System accuracy	TRE $<5 \hbox {mm}$	TRE $<3 \hbox {mm}$
R6. Surgical display	Intuitive GUI with 3D representation of tumour and surrounding anatomical structures (e.g. cranial nerves) integrated with navigated neurostimulation points and 3D ultrasound reconstructions	Ibid Fully integrated neurostimulation recordings and automatically generated 3D ultrasound image reconstructions
R7. Ultrasound visualisation	Navigated US using rigid registration method enabling 3D reconstruction of image in conventional axial, sagittal and coronal planes	Image-based non-rigid registration method enabling automated real-time image reconstructions
R8. Neurostimulation recordings	Neurostimulation points recorded by operator and parameters added manually	Fully integrated neurostimulation with position and parameters automatically recorded and displayed on images and 3D model
R9. Imaging Rate	Imaging rate which does not impede surgical workflow (minimum 7 FPS)	Video-rate imaging of at least 25 FPS

*US* ultrasound, *TRE* target registration error; *GUI* graphical user interface, *FPS* frames per second

The intraoperative system should be straightforward to use without the need for technical support (**R1**). System components should comprise standard, commercially available hardware, and assembly of non-sterile components should ideally be completed within 15 min (**R1**). To ensure surgical sterility and safety is maintained throughout, intraoperative sterile components should not be altered from their designated use and the introduction of any additional hardware and functional capabilities must not compromise surgical safety or sterility (**R2**). To minimise interference with the routine surgical workflow, our minimum design requirement stipulated that probe calibration should be completed within 1 min, but calibration-free (i.e. factory/laboratory calibration only) instruments should be a target requirement for future systems (**R3**). In a preliminary study, we tested the BK ultrasound system in isolation in patients undergoing skull base surgery and observed that a fixed image depth of 4.5 cm could usually be used to image the surgical scene. Consequently, as a minimum requirement, we decided to set a fixed depth for image calibration (i.e. the affine transform between the US image and the tip of the probe) and automated variable calibration was set as a desirable future target requirement (**R4**). A Target Registration Error (TRE) of $<5 \hbox {mm}$ was set as a minimum requirement for the navigated ultrasound reconstruction with a TRE of $< 3\,\hbox {mm}$- comparable to the TRE of current commercial neuronavigation systems, set as a target requirement (**R5**).

The system’s end-user interface should be intuitive to use displaying data in a user-friendly manner in real time and should enable the user to easily switch between viewing modes as required (**R6**). Navigated US should enable 3D reconstruction of the imaging data in conventional axial, sagittal and coronal planes with a target requirement of image-based non-rigid registration methods enabling automated real-time US image reconstructions (**R7**). Ideally, the system should be capable of importing the neurophysiological data in real time, pairing it with tracked data points for viewing on the surgical display (**R8**). The minimum imaging rate for reconstructed ultrasound images must be fast enough to provide real-time information suitable for surgical decision making without interfering with the surgical workflow (**R9**). Based on speed of processing in the human visual system, an image visualisation rate faster than 7 frames per second (FPS) is desired [26]; however, a system capable of providing video-rate imaging (approximately 25 FPS to 30 FPS) should be the target requirement of such a system.

## System design

### System configuration

**Fig. 1:**
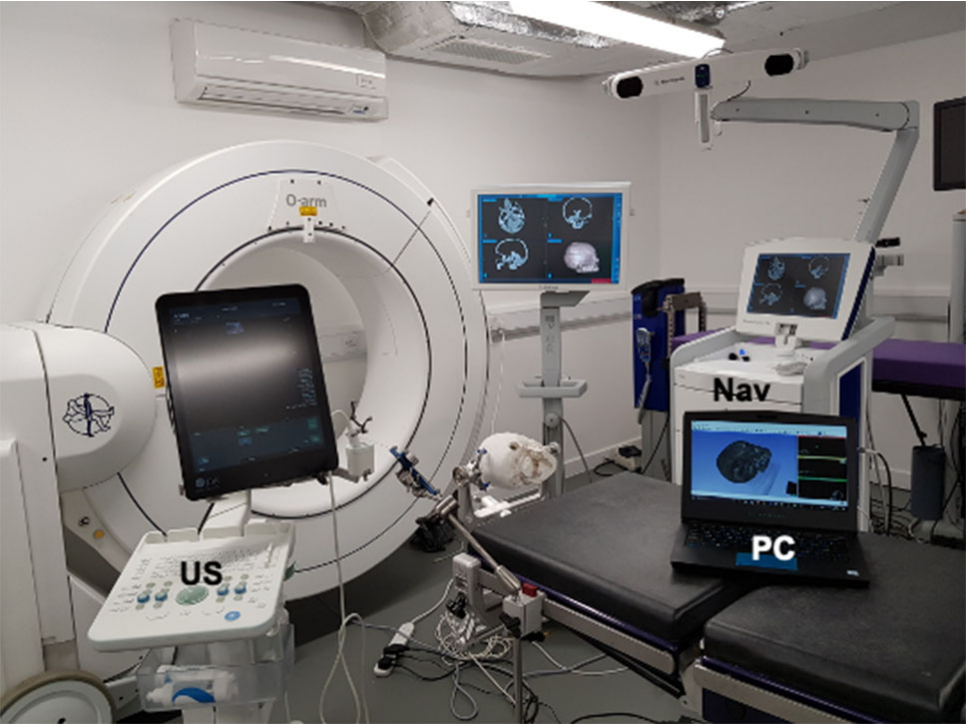
System setup—Nav: Medtronic Stealthstation, US: BK 5000 Ultrasound system, PC: Laptop

System hardware included a commercial intraoperative ultrasound system (BK 5000 Ultrasound system), neuronavigation system (Medtronic Stealthstation) and a standard PC as illustrated in Fig. 1. By only using existing hardware within its intended use, the SBN system complied with all relevant surgical safety requirements including sterility and electrical standards. The additional pieces of hardware needed for this prototype system included a network switch and interconnecting cables. A laptop placed on a small surgical trolley could be positioned conveniently within the OR as directed by the surgical team. The system enabled various different imaging and monitoring modalities to be integrated into a single user-friendly navigation system. Optical tracking of both the neurostimulation probe and US transducer was achieved with the Medtronic SureTrak^TM^ universal instrument adapter system.

**Fig. 2:**
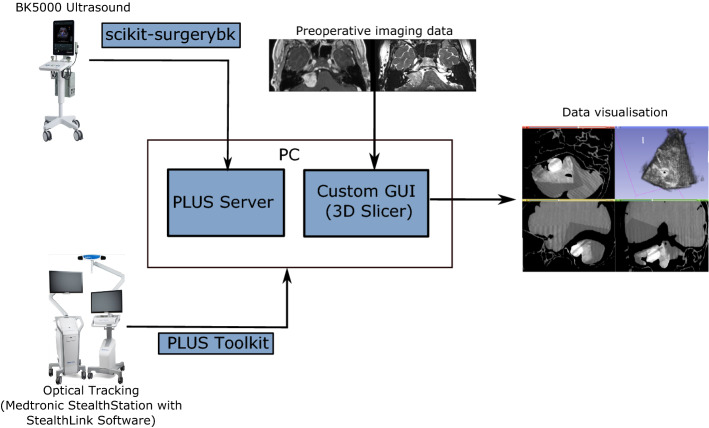
System architecture. Medtronic Stealthstation with StealthLink software and BK5000 Ultrasound hardware used to stream data to PC via PLUS Toolkit and Scikit-SurgeryBK and PLUS Server, respectively. Custom GUI built using 3D Slicer software

To enable streaming of data between devices, a local network was established connecting a BK5000 Ultrasound, Medtronic Stealthstation Optical Tracker and PC (Fig. 2). Optical trackers were attached to the ultrasound probe and neurostimulator. Ultrasound data were streamed across the network using the Scikit-SurgeryBK library [25], and the PLUS Toolkit [11] (https://plustoolkit.github.io/) was used to stream tracking and model data from a StealthLink enabled Medtronic Stealthstation. Ultrasound and tracking data were received on the client PC using the PLUS Toolkit in the OpenIGTLink format (http://openigtlink.org/). The BK 5000 Ultrasound system is a 2D B-mode ultrasound system with 3D reconstruction achieved in our system via calibration using ultrasound and tracking data within the PLUS Toolkit. A frame rate of approximately 25 FPS with a clinically acceptable latency was achieved using this configuration.

In terms of software design, we exploited existing established open-source software whenever relevant to enable rapid prototyping and provide a framework into which new functionality could more easily be added. PLUS Toolkit, OpenIGTLink and Slicer are well-established open-source tools in the IGS field and provided the bulk of the functionality needed for data acquisition, ultrasound reconstruction and data visualisation. Proprietary software from Medtronic was needed to stream data from the Stealthstation. While PLUS provides functionality for streaming data from the BK5000, the decision was made to use an alternative Python implementation (Scikit-SurgeryBK) for US streaming, as it was preferable to control streaming from the main Python codebase.

**Fig. 3:**
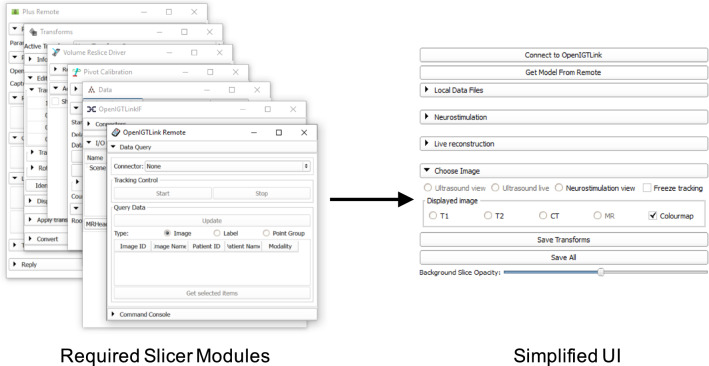
Simplified “Slicelet” user interface. A schematic illustration of the “Slicelet” system whereby extraneous GUI components were removed to provide a simplified workflow for use in the OR. The “Slicelet” combines functionality from seven different Slicer modules into a single UI panel, greatly simplifying the clinical workflow by automating several tasks, removing extraneous components and eliminating the need to manually switch between and configure different modules

A custom end-user interface was created using 3D Slicer, as a *slicelet* (https://www.slicer.org/wiki/Documentation/Nightly/Developers/Slicelets) where extraneous GUI components were removed and setup/communication with external devices was automated, providing a greatly simplified workflow for use in the OR (Fig. 3). Features of the slicelet included display of preoperative MR/CT scans with volume reconstructions of key structures, real-time overlay of ultrasound data and tool locations such as the neurostimulation probe with means to enter relevant neurophysiological data and a simplified pivot calibration process (as described in “Calibration” section) for tracked tools and volume reconstruction of ultrasound data (Fig. 2).

### Calibration

Position tracking of the neurostimulation probe and ultrasound transducer was calibrated at the start of each case using the pivot calibration algorithm provided as part of SlicerIGT software [28]. Pivot calibration involved using the tracked Stylus tool to determine the tip of each individual instrument relative to the SureTrak^TM^ marker and was performed by the operating surgeon using sterile instruments within the surgical field. The calibration procedure was a two-step process that involved pivoting and then spinning the tracked instrument around a fixed point for 15 s each in turn. Following each movement, the root-mean-square error (RMSE) value was computed. Typically, the RMSE for pivot and spin calibration should be less than 1 mm (http://www.slicerigt.org/wp/user-tutorial/ Tutorial U11- Pivot Calibration). As the tracker is securely fixed to the tool, once calibration has been performed, the transformation matrix between the stylus tip and the tracker remains constant.

**Fig. 4:**
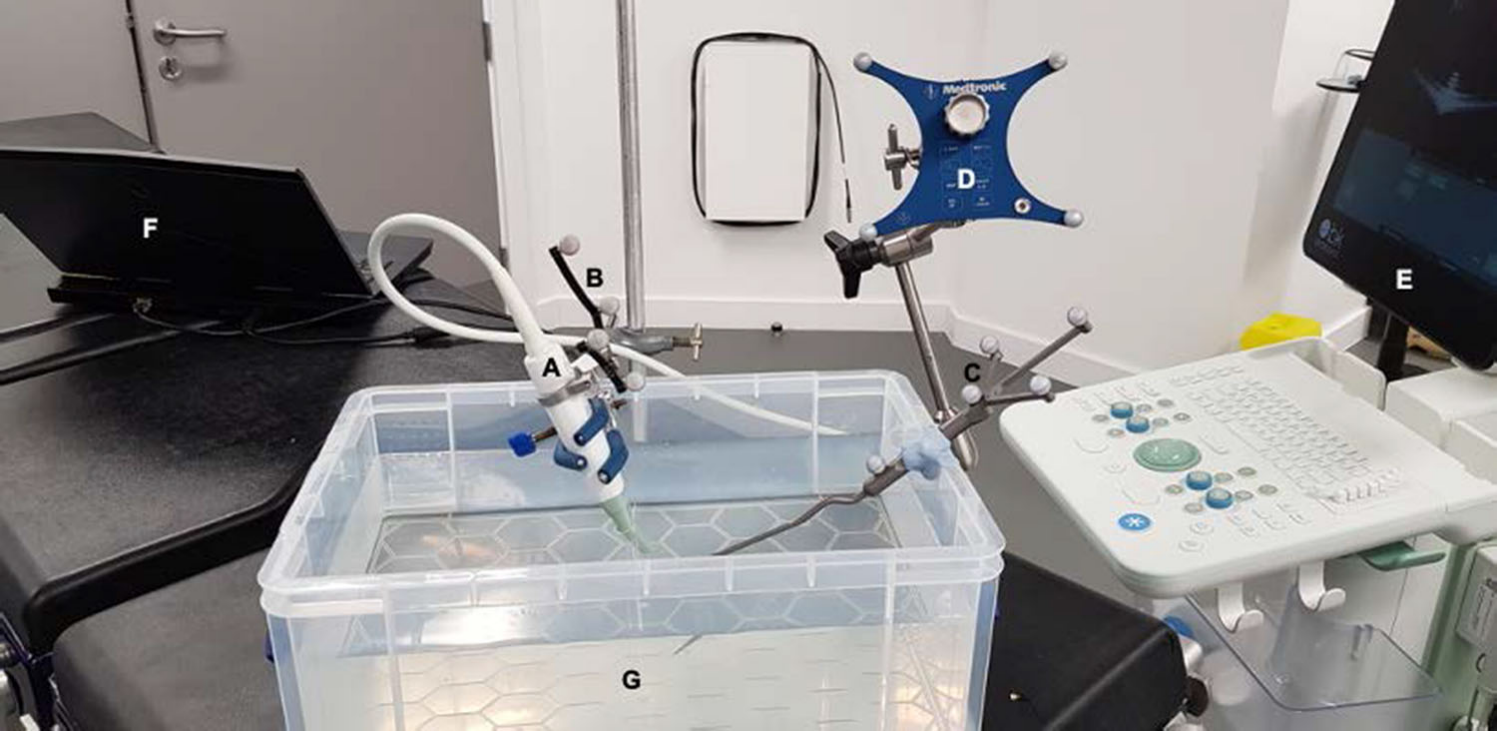
Ultrasound image calibration: spatial calibration method. **a** Ultrasound transducer (clamp for illustration purposes only); **b** Medtronic SureTrak^TM^ optical tracking marker; **c** Medtronic Stylus; **d** Medtronic reference frame; **e** BK 5000 Ultrasound machine; **F** computer; **G** water tank

Temporal and spatial ultrasound image calibrations were performed using the PLUS Freehand tracked ultrasound calibration (fCal) application [11]. Ultrasound image calibrations were performed in the laboratory using a water tank under strict experimental conditions (Fig.  4). Temporal calibration was acquired by tracking the ultrasound transducer up and down with a periodic motion while imaging the bottom of the water tank. Spatial calibration was performed using the stylus-aided calibration toolbox and involved imaging and registering the stylus tip in multiple locations within the ultrasound image. The transformation matrix was subsequently saved for use during surgery.

## Accuracy and workflow testing: methods

### Laboratory testing of system accuracy

An abstract polyvinyl alcohol cryogel (PVA-c) phantom model was used to test the accuracy of the system’s ultrasound navigation and reconstruction. The phantom consisted of two spherical 15-mm-diameter tumour-mimicking spheres embedded within parenchyma-mimicking tissue and was manufactured according to previously published methodology [4, 9, 20]. For the tumour spheres, talcum powder was added to the base PVA mixture to act as an ultrasound contrasting agent. The tumour spheres underwent an additional 12-hour freeze–thaw cycle before they were suspended into the parenchyma-mimicking tissue to complete a further two 12-hour freeze–thaw cycles.

**Fig. 5:**
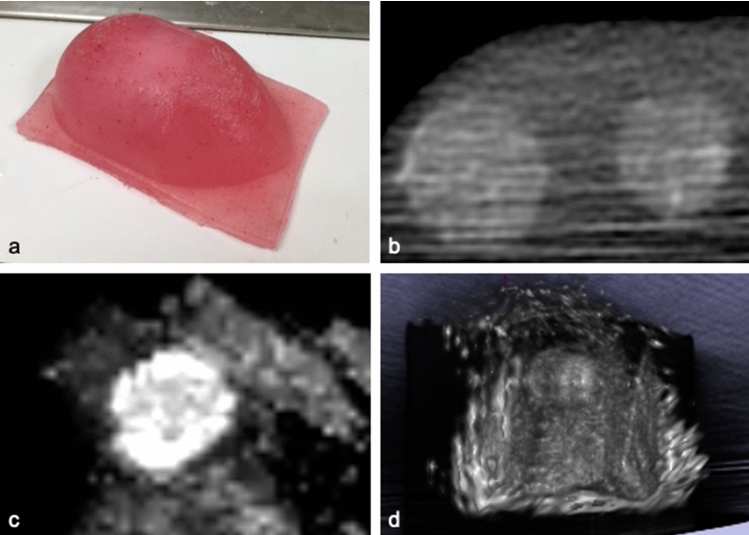
Validation of the system’s ultrasound navigation and reconstruction using a multi-modal polyvinyl alcohol (PVA) phantom. **a** Polyvinyl alcohol phantom; **b** volumetric 3D X-ray image of phantom obtained with the Medtronic O-arm; **c** reconstructed ultrasound image obtained with an intraoperative burr hole ultrasound transducer (N11C5s) connected to a BK 5000 Ultrasound system; **d** volumetric reconstruction of ultrasound data

The phantom was imaged with a Medtronic O-arm^TM^ to provide a 3D volumetric X-ray contrast image for registration. Using the SBN system, phantom data were uploaded to the Medtronic Stealthstation and registered with the model using a surface-matching trace technique. An intraoperative BK burr hole ultrasound transducer (N11C5s) connected to a BK 5000 Ultrasound system was calibrated using the method described above, and volumetric ultrasound data were acquired (Fig.  5).

Tumour spheres were segmented on the registered ultrasound and X-ray images using an intensity threshold. The binary segmentations were converted to closed surface meshes using NifTK’s [7] Surface Extractor plugin, which uses VTK’s [23] implementation of the marching cubes algorithm. Registration errors were measured using Dice scores, and the TRE was calculated between the two centres in 3D. Sphere fitting was done using the SciKit-Surgery-Sphere-Fitting application [24], part of the SciKit-Surgery project [25]. SciKit-Surgery-Sphere-Fitting fits a sphere of fixed radius to the surface point data, using least squares optimisation implemented in the SciPy library [30]. Dice scores on the fitted spheres were calculated using the two_polydata_dice function from the SciKit-SurgeryVTK library [6], and the TRE was calculated between the two centres in 3D.

### Clinical simulation to test workflow integration

**Fig. 6:**
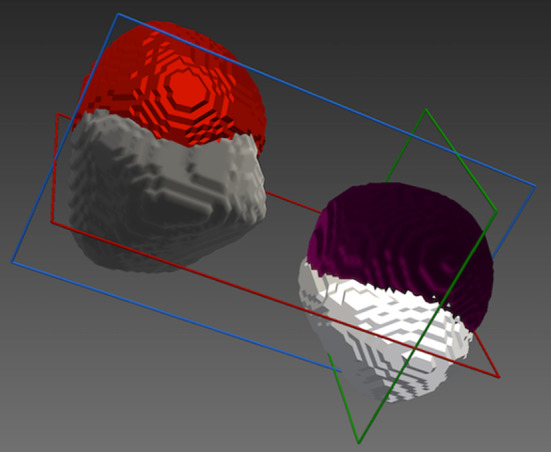
Segmentations of the tumour spheres obtained using registered volumetric ultrasound and 3D X-ray images. *Maroon/Red*: Segmentations of 3D X-ray images; *White/Grey*: Segmentations of reconstructed volumetric ultrasound images; tumour sphere 1: maroon/white; tumour sphere 2: red/grey

A patient-specific PVA-c phantom model comprising the skull, brain and tumour created with tissue-mimicking ultrasound and X-ray properties was used to simulate the use of the navigation system in a clinical operating room. The corresponding detailed phantom manufacturing protocol can be found in our previous work [14]. The time taken to set up the system and to perform probe calibration was recorded, and clinician feedback was obtained regarding the clinical utility and accuracy of the system (Fig. 6).

## Accuracy and workflow testing: results

The TRE between the centre of the fitted spheres was 3.82 mm and 4.41 mm for tumour spheres #1 and #2, respectively. Dice scores were 0.64315 and 0.60275, respectively.

**Fig. 7:**
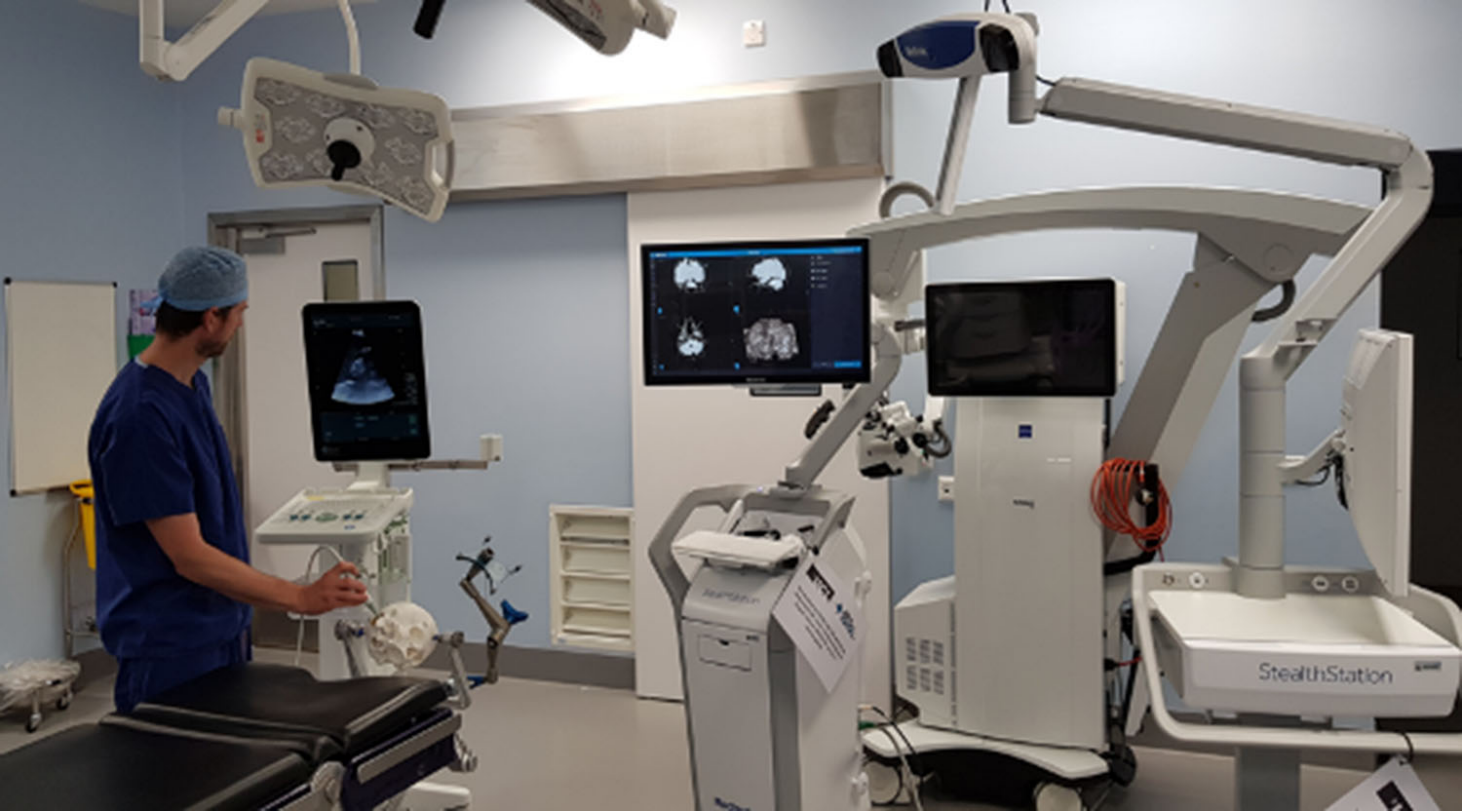
Intraoperative simulation of SBN system in a clinical operating room using a patient-specific phantom model

**Fig. 8:**
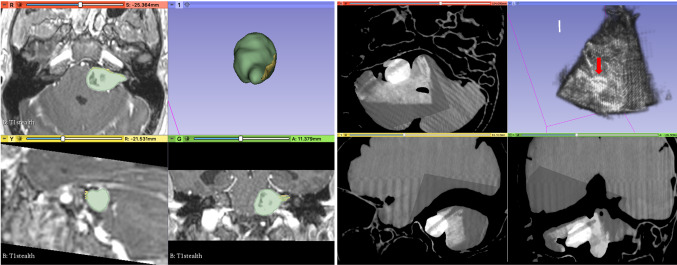
Intraoperative simulation of 3D Ultrasound reconstruction using a patient data and a patient-specific phantom model. **a** Illustration of patient MRI data using the system including an overlay of the tumour and nerve on the MRI data and a separate 3D model of those structures. Green: tumour, Yellow: nerve **b** system display of CT scan of phantom model with overlaid 3D reconstructed ultrasound (US) data. Volumetric representation of the US data is displayed in the top right panel. *Red arrow*: tumour

The integrated navigation system was also tested in a clinical operating room (Figs. 7, 8). Trained clinical staff correctly assembled the system hardware and completed initial set-up in 10 min  19 s. Assembly of the system’s sterile components was completed correctly in 1 min 22 s, and intraoperative probe calibration was completed in 43 s. Clinical evaluation of the system was undertaken independently by 2 consultant neurosurgeons using a patient-specific PVA-c phantom model of a patient with a vestibular schwannoma undergoing surgical resection via a simulated retrosigmoid craniotomy. Both neurosurgeons considered the system to be highly useful, with an intuitive display and clinically acceptable accuracy (Fig. 8). The desired functionality of creating a fully integrated display system combining preoperatively acquired imaging data with real-time intraoperative 3D ultrasound and navigated intraoperative neurophysiological stimulation points was successfully simulated on the phantom model.

## Discussion and conclusions

We present an integrated intraoperative navigation system tailored to skull base neurosurgery with the ability to incorporate (1) preoperative structural MR and CT imaging and 3D volume reconstructions of the tumour and surrounding anatomy (e.g. facial nerve), (2) neurophysiological monitoring and stimulation and (3) live reconstructed 3D ultrasound. The system was built around commercially available CE-marked hardware to facilitate clinical translation although additional proprietary software/licence for streaming the data out of the commercial devices was required. All other system’s software components including the 3D Slicer platform, PLUS and Scikit-SurgeryBK software libraries are open source.

Other commercially available navigated ultrasound systems are available (e.g. Brainlab and Esaote ultrasound systems), but neither system provides the capability to fully integrate multi-modal intraoperative data streams such as neuromonitoring and stimulation. A number of research-orientated intraoperative navigation systems capable of integrating real-time ultrasound have previously been reported [2, 8, 16, 19], but most of these are built around general medical imaging platforms rather than being designed for intraoperative image-guided neurosurgery. Despite the fact that the IBIS and CustusX platforms are dedicated to IGS with a user interface tuned towards intraoperative use [2, 16], the available documentation made it difficult to integrate these systems with our existing clinical hardware and adding other non-IGS functionalities was not trivial. Consequently, we designed an integrated navigation system that can be used with any type of clinical hardware. We chose to build the system using 3D Slicer software an open-source software package with excellent documentation, enabling us to draw upon the resources of a platform with established large feature sets and a well-defined quality process.

Our prototype research system met all of the minimum requirements stipulated in Table 1. The surgically safe system complied with standard sterile practices (**R1,2**). Using several stand-alone medical devices during a surgical procedure is common practice. Because our system does not alter or change the intended use of any of the individual pieces of hardware, we substantially reduce the risk of using our integrated research system in an ethically approved clinical study. It was easy and quick to assemble (**R1,2**), and intraoperative probe calibration took less than a minute (**R3**). Following preliminary in vivo clinical studies to ascertain the commonest ultrasound imaging depth used in skull base surgery, the current system was calibrated at a fixed imaging depth of 4.5 cm, but future work is underway to enable a more robust automatic variable image calibration (**R4**). Video-rate imaging of 25 FPS was achieved, as per the target requirement (**R9**). Laboratory testing demonstrated comparable system accuracy levels to previously reported research systems (**R5**) [2, 16], and in clinical testing, surgeons reported the system to be clinically acceptable (**R5**). Nevertheless, the TRE achieved in our phantom experiment was slightly higher than our target requirement of $< 3 \hbox {mm}$. Future work will investigate what error sources contribute to the current TRE and look at ways to reduce the most significant error sources. We currently believe that calibration of the ultrasound probe is a significant source of error, so future work will investigate using alternative calibration methods, for example, phantomless auto-calibration [27].

In the system’s present version, neurostimulation data must be entered manually; however, it has the potential to fully integrate with standard neuromonitoring systems (e.g. inomed neuromonitoring systems; https://www.en.inomed.com/products/intraoperative-neuromonitoring-ionm/) to enable continuous and automatic recording and display (**R8**). The system’s user interface was felt to be “clear” and “intuitive”, but further refinement in collaboration with commercial partners is currently underway to improve the GUI’s aesthetic appearance (**R4**). Future work aims to optimise the system further by addressing the remaining target requirements. Alternative calibration methods will be evaluated, and different software will be tested in order to improve temporal calibration between the optical tracking and ultrasound data sources. By making this software open source, we are also enabling others in the research community to test and build upon this work. The system’s architecture, built around other open-source platforms, increases its compatibility with various commercial systems, thus extending its potential use beyond neurosurgery alone.

## Footnotes

1 http://www.brainlab.com; Brainlab AG, Munich, Germany.

2 http://www.esaote.com/ultrasound; Esaote, Genoa, Italy.

3 https://www.gov.uk/guidance/regulating-medical-devices-in-the-uk.
